# Frequency of Tommy John Surgery in NCAA Division I College Pitchers Versus Weather Conditions

**DOI:** 10.1177/23259671241311601

**Published:** 2025-01-21

**Authors:** Sabrina M. Pescatore, Sterling J. DeShazo, William M. Weiss

**Affiliations:** † The University of Texas Medical Branch at Galveston, Galveston, Texas, USA; Investigation performed at The University of Texas Medical Branch at Galveston, Galveston, Texas, USA

**Keywords:** elbow, physical therapy/rehabilitation, softball/baseball, Tommy John surgery, ulnar collateral ligament

## Abstract

**Background:**

Ulnar collateral ligament reconstruction (UCLR) is a common elbow procedure in baseball pitchers. Previous studies of Major League Baseball pitchers identified the weather as a potential risk factor, as warmer climates enable more annual playing time and increase overuse injury risks.

**Purpose:**

To determine whether weather conditions play a role in UCLR rates and timing for National Collegiate Athletic Association (NCAA) Division I (D1) collegiate pitchers in the United States.

**Study Design:**

Cross-sectional study; Level of evidence, 3.

**Methods:**

A total of 320 NCAA D1 college baseball pitchers who underwent UCLR surgery between July 1, 2015, and June 30, 2022, were analyzed. Pitcher college climates were categorized as warm or cold based on their location relative to the 33rd parallel line in North America. A 2-sample independent *t* test was used to compare the mean UCLR rate for pitchers in warm versus cold climates. The incidence rate difference and incidence rate ratios by state and pitcher year were calculated and evaluated. The chi-square test and Poisson Regression were used to evaluate associations between pitcher year and high school pitching climate.

**Results:**

Among 320 total UCLRs, warm-state pitchers had a higher mean UCLR rate compared with cold-state pitchers (*P* = .0001). The highest number of UCLRs in warm states occurred during the sophomore year (n = 57), while the highest number of UCLRs in cold states occurred during the junior year (n = 63). Freshmen, sophomore, and senior warm state pitchers had significantly higher (incidence rate ratios [IRR] and incidence rate difference [IRD]) rates and likelihood of UCLR than their cold state counterparts (freshmen *P*_IRD_ = .0025, *P*_IRR_ = .0032; sophomore: *P*_IRD_ = .0002, *P*_IRR_ = .0003, senior: *P*_IRD_ = .0123, *P*_IRR_ = .0159). Underclassmen (freshmen and sophomores) pitchers who threw in warm high school climates had a 1.4 times higher rate of UCLRs than underclassmen pitchers from cold high school climates (*P* = .041).

**Conclusion:**

NCAA D1 college baseball pitchers who play in warm climates undergo UCLR surgery significantly more often and significantly earlier in their collegiate careers than pitchers playing in cold climates.

Ulnar collateral ligament (UCL) reconstruction (UCLR), or Tommy John surgery, is an increasingly common elbow procedure among overhead motion athletes such as baseball players.^11,17^ The current literature suggests that UCL injuries are a product of overuse from repeated overhead motions.^4^ In baseball pitchers, the UCL is put under significant valgus stress with a strong torque force, which leads to excessive stress on the UCL repeatedly over time.^16,24,33^ UCLR rehabilitation time and return to play can be lengthy, with athletes returning to sport anywhere between 10 and 18 months on average.^12^ In addition, UCLR surgery is frequently accompanied by several postoperative complications—including elbow stiffness (13%), ulnar nerve neuropathy (26%), and other issues that can impact recovery.^5,6,8,32^

Although there is some inconsistency in the research, risk factors—such as inadequate warm-up, high pitch velocity, greater mean pitch count, fewer days between games, and limited repertoire of pitches—have all been implicated in increased risk for UCLR.^26,30,34^ Another potential risk factor is the weather, with some evidence suggesting that Major League Baseball (MLB) pitchers in warmer climates may be at a higher associated risk for UCLR; however, this risk factor has not previously been associated with collegiate baseball pitchers.^13,27^ A recent survey of National Collegiate Athletic Association Division I (NCAA D1) coaches did not find a link between the team’s geography and the coaches understanding of and prevention efforts for UCL injuries.^2^ This suggests that misconceptions about these key risk factors likely contribute to improper injury-prevention care at a national level and geographic differences are unlikely to significantly impact attention to warm-up, pitching velocities, pitch counts, rest days, and pitch types for NCAA D1 pitchers.^2^

Given the lack of regional differences in UCLR understanding and prevention among NCAA D1 coaches and the high morbidity of UCL injuries, investigating the impact of geography and weather conditions on UCLR risk in D1 collegiate pitchers could provide new insights and allow for the implementation of more effective climate-specific injury prevention strategies. This study aimed to evaluate the impact of weather conditions on the associated risk and timing for UCLR surgeries in D1 collegiate pitchers. We hypothesized that D1 pitchers in warmer geographical climates would be more predisposed to UCLRs than their cold-climate counterparts.

## Methods

**Figure 1.**
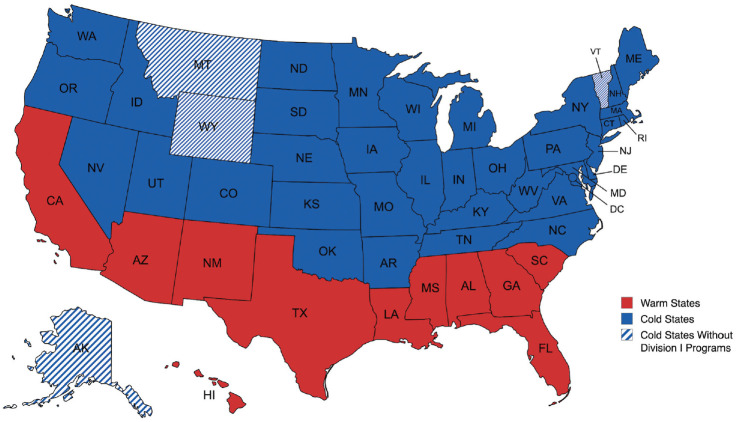
Categorization of warm states versus cold states relative to the 33^rd^ parallel.^22^

Based on the demarcation line previously defined by Erickson et al^13^ in their study of MLB pitchers, United States (US) states south of the 33rd parallel in North America were categorized as warm-climate states, while US states north of the 33rd parallel were categorized as cold-climate states.^13^ This determination was made due to states south of the 33rd parallel maintaining daily average temperatures during the coldest month of the year (January) that were above freezing. Pitchers playing in these environments could theoretically throw year-round, accumulating more overall pitching time. As a result, 11 warm-climate states and 40 cold-climate states—including Washington, District of Columbia—were identified, with 4 of the cold states not having D1 college baseball programs during our study period (Figure 1).

The number of UCLR surgeries for D1 collegiate pitchers and their respective academic year was obtained from the Tommy John Surgery List, a publicly available, online spreadsheet of UCLR injury statistics.^29^ There was a total of 320 D1 college baseball pitchers identified who had UCLR surgery between July 1, 2015, and June 30, 2022, representative of the 2015-2016 to 2021-2022 collegiate baseball seasons. For each injured pitcher, their respective college, college location by state, and the state where they played baseball in high school were documented (via internet searches) and categorized as warm or cold. Institutional review board approval was not required since this study used only publicly available records and did not involve interaction or intervention with human subjects.

The number of D1 baseball programs per state and the number of players was determined using the NCAA Sports Sponsorship and Participation data for the 2015-2016 to 2021-2022 seasons.^31^ The mean number of D1 baseball teams per year was 296 teams: 107 from warm states and 189 from cold states. The mean number of players per team per season was 37.0381 or a total of 76,706 players. The number of total players calculated was cross-referenced with the NCAA-reported data total to ensure accuracy. The number of D1 pitchers was estimated via the NCAA reported average of 13.5 pitchers per team.^18,25,31^ Therefore, the estimated^18,25,31^ total number of pitchers was 27,959.

Two-sample independent *t* tests were used to compare the mean temperatures between warm-climate states versus cold-climate states and to compare the mean UCLR rates for pitchers in warm- versus cold-climate states. Because of the lack of available data on the exact number of freshmen, sophomore, junior, and senior pitchers per team per year, we utilized incidence rate ratios (IRR) and incidence rate differences (IRD) to quantify the incidence rate and the absolute difference in UCLR incidence rates between pitcher academic year and respective climate. In addition, a chi-square test and Poisson regression were used to evaluate possible associations with high school baseball climate pitched in compared with UCLRs by pitcher academic year. Pitchers were combined into underclassmen (freshmen and sophomores) and upperclassmen (juniors and seniors) to increase the power of these analyses. *P* < .05 was considered significant for all comparisons.

## Results

**Figure 2.**
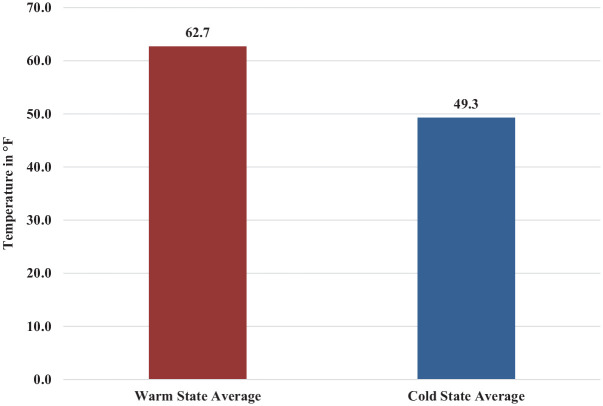
The mean temperature (in °F) of warm-defined states at or below the 33rd parallel versus cold-defined states above the 33rd parallel.

The mean annual temperature from 2015-2022 for each state with a D1 baseball program was identified and averaged based on their warm or cold classifications.^1^ The mean annual temperature for warm states was 62.7°F and the mean annual temperature for cold states was 49.3°F, with a difference of 13.4°F when compared (Figure 2). The mean temperature in warm versus cold states was compared using a 2-sample independent *t* test, and the results were significant (*P* = .0001).

**Figure 3.**
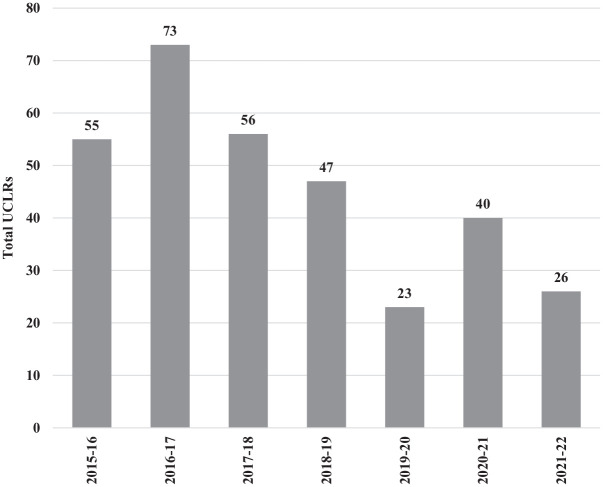
Total UCLRs in D1 college pitchers per season between July 1, 2015, and June 30, 2022. D1, division 1; UCLR, ulnar collateral ligament reconstruction.

Of the 27,959 total D1 pitchers estimated in the 2015-2016 to 2021-2022 seasons, 10,139 (36.3%) played at colleges in warm states, while 17,820 (63.7%) played at colleges in cold states. Of the 320 total D1 college pitchers who underwent UCLR surgery, 158 (49.4%) occurred in a warm state and 162 (50.6%) occurred in a cold state. The 320 D1 college pitchers who underwent UCLR surgery during the 2015-2016 to 2021-2022 seasons are represented in Figure 3 by season of occurrence.

**Figure 4.**
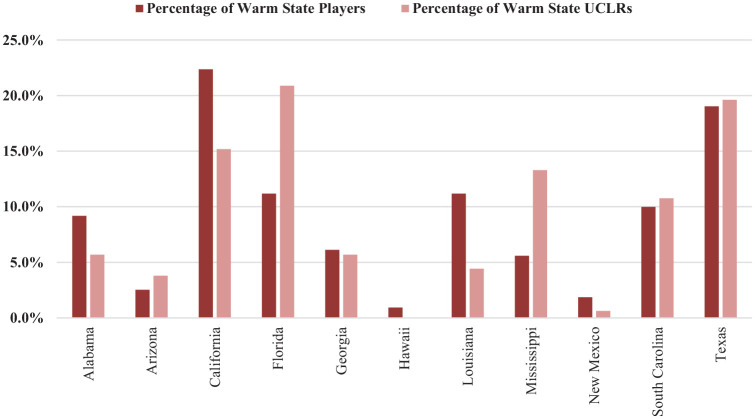
Percentage of warm state total pitchers versus percentage of warm state pitchers who sustained UCLRs for the 2015-16 to 2021-22 seasons.

**Figure 5.**
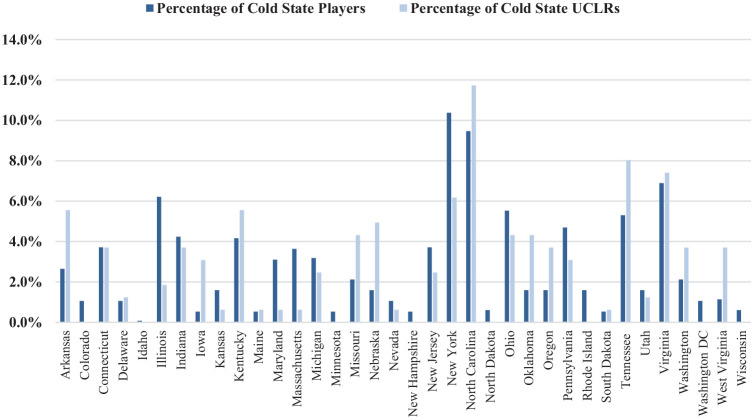
Percentage of cold state total pitchers versus percentage of cold state pitchers who sustained UCLRs for the 2015-16 to 2021-22 seasons.

See Figures 4 and 5 for the distribution of estimated average total baseball pitchers and actual pitcher-sustained UCLRs by warm or cold state. The rate of pitchers undergoing UCLRs from warm states was significantly higher (0.0156 per pitcher) than the rate from cold states (0.0091 per pitcher) (*P* = .00011).

The highest number of annual UCLRs for pitchers in warm states occurred during the sophomore year (n = 57; 36.1%), whereas the highest number of annual UCLRs for pitchers in cold states occurred during junior year (n = 63; 38.9%) (Table 1). Warm states had more UCLRs for freshmen and sophomore pitchers combined (62.7% warm vs 53.7% cold), while cold states had more UCLRs for juniors and senior pitchers combined (46.3% cold versus 37.4% warm).

**Table 1:** UCLRs by Pitcher Year and Warm Versus Cold Weather State^*a*^

Pitcher Year	Total UCLRs		Warm state UCLRs		Cold state UCLRs	
Freshmen	80	25	42	26.6	38	23.5
Sophomore	106	33.1	57	36.1	49	30.2
Junior	105	32.8	42	26.6	63	38.9
Senior	29	9.1	17	10.8	12	7.4
Total	320	100	158	100	162	100

a Data presented as numbers and percentages. UCLRs, ulnar collateral ligament reconstructions.

Rates were calculated for warm and cold state pitchers by their respective college years using pitcher totals (warm state = 10,139; cold state = 17,820) and compared for differences and incidence ratios (Table 2). Warm state freshmen, sophomore, and senior pitcher UCLR ratios and incidence were significantly different than cold state freshmen pitcher UCLRs (freshmen: *P*_IRD_ = .0025, *P*_IRR_ = .0032; sophomore: *P*_IRD_ = .0002, *P*_IRR_ = .0003, senior: *P*_IRD_ = .012, *P*_IRR_ = .0159). There was no difference in warm state versus cold state junior pitcher UCLR ratios and incidence.

**Table 2:** Total counts, Overall Rates, Incidence Rate Differences, and Incidence Rate Ratios of UCLRs^*a*^

Pitcher Year	UCLR Warm	UCLR Cold	UCLR Rate Warm	UCLR Rate Cold
Freshmen	42	38	0.0041	0.0021
Sophomore	57	49	0.0056	0.0027
Junior	42	63	0.0041	0.0035
Senior	17	12	0.0017	0.0007
Pitcher Year	Incidence Rate Difference		95% CI	*P* (*P*_IRD_)
Freshmen	0.0020		0.0007 to 0.0033	.0025
Sophomore	0.0029		0.0014 to 0.0044	.0002
Junior	0.0006		−0.0009 to 0.0022	.4257
Senior	0.0010		0.0002 to 0.0018	.0123
Pitcher Year	Incidence Rate Ratio		95% CI	*P* (*P*_IRR_)
Freshmen	1.9427		1.2226 to 3.0959	.0032
Sophomore	2.0446		1.3711 to 3.0589	.0003
Junior	1.1718		0.7736 to 1.7589	.4261
Senior	2.4900		1.1207 to 5.7141	.0159

a UCLR, ulnar collateral ligament reconstruction.

Most of the injured pitchers who grew up playing high school baseball in cold or warm states played college baseball in the same respective climate (125 from a cold-climate high school to a cold-climate college; 130 from a warm-climate high school to a warm-climate college). A total of 28 pitchers played high school baseball in a cold state but played college baseball in a warm state, while 37 pitchers moved from a warm high school state to play in a cold college state (Table 3). Using a Poisson regression analysis and a chi-square test, we analyzed whether high school climate had an association with overall UCLR or was a predictor for earlier UCLRs in pitchers’ collegiate careers. There was a total of 167 pitchers from warm-climate high schools and 153 pitchers from cold-climate high schools. The chi-square test of independence revealed that although pitchers from warm-climate high schools had a higher likelihood of undergoing UCLR in college than pitchers from cold-climate high schools, there was no statistically significant difference (χ^2^ = 2.972, *P* = .085). To investigate whether high school climate was associated with earlier instances of UCLR in pitchers’ collegiate careers, pitchers from warm- and cold-climate high schools were divided into underclassmen and upperclassmen and analyzed. Poisson regression revealed that underclassmen pitchers from warm-climate high schools (n = 107) had a 1.354 times higher rate of UCLR compared with underclassmen from cold-climate high schools (n = 79) (*P* = .041). However, upperclassmen pitchers from cold-climate high schools (n = 74) had a 0.598 times higher rate of UCLR compared with upperclassmen from a warm-climate high school (n = 60) (*P* = .040).

**Table 3:** UCLRs by Pitcher Year and Climate^*a*^

High School Versus College Climate	Freshmen UCLRs	Sophomore UCLRs	Junior UCLRs	Senior UCLRs	Total
Cold-climate high school to cold-climate college	29	34	54	8	125
Warm-climate high school to warm-climate college	32	51	34	13	130
Cold-climate high school to warm-climate college	10	6	8	4	28
Warm-climate high school to cold-climate college	9	15	9	4	37
Total	80	106	105	29	320

a UCLRs, ulnar collateral ligament reconstructions.

## Discussion

Our results indicate that D1 collegiate pitchers are significantly more likely to undergo UCLR surgery if they pitch in warmer states. Pitchers who threw in warm weather states averaged 0.0156 UCLRs per season for the 2015-2016 to 2021-2022 seasons, while pitchers who threw in cold weather states averaged 0.0091 UCLRs per season (*P* = .00011). A potential reason for these results could be that pitchers in warmer environments may have longer in-season practices and shorter off-seasons because of better annual weather conditions. The difference between warm state mean temperature (62.7°F) and cold state mean temperature (49.3°F) was also significant (*P* < .0001).

The physiologic effects of temperature on tissue and muscular changes could also be contributing to these findings. Existing research has shown that warmer weather enhances muscle contraction force and speed, improves ligament and tendon flexibility and elasticity, increases range of motion, and reduces the incidence of acute stiffness-related injuries.^14,15,23,28^ Higher temperature climates may induce more elasticity and flexibility of the tissues, thereby allowing pitchers to throw at higher velocities via an increased range of motion and greater torque.^14,23^ However, although beneficial in the short term, these biomechanical changes ultimately put greater strain on the UCL ligament in cases of repeated high-velocity use. Therefore, they may contribute to increased occurrences and earlier instances of UCLRs observed in warm climate pitchers.^14,28^

After trending up from 2015-2016 to 2017-2018, pitcher UCLRs had a negative trend from 2018-2019 to the 2021-2022 seasons, with an inflection point in the 2019-2020 season. We believe this is attributable to the coronavirus disease 2019 (COVID-19) pandemic, which resulted in shorter preseasons, shorter regular seasons, and in some cases, canceled seasons in the spring of 2020. States with the most pitcher UCLRs during the 2015-2016 to 2021-2022 seasons were Florida (34), Texas (31), and California (23), respectively. The number of UCLRs in pitchers from these states comprises 28% of all pitcher UCLRs during the 2015-2016 to 2021-2022 seasons, while they only represent 19% of total D1 baseball pitchers in the nation. Overall, there was a disproportionate number of UCLRs in warm states (49.4%) when considering that warm states comprise only 36.3% of total D1 baseball pitchers.

On average, pitchers who play in warm climates also underwent UCLRs earlier in their collegiate careers compared with pitchers who play in cold climates. Between the years 2015-2022 for all climates, 25% of total UCLRs were freshmen, 33.1% were sophomores, 32.8% were juniors, and 9.1% were seniors. Interestingly, freshmen in warm climates comprised 26.6% of the total warm climate UCLRs and sophomores comprised 36.1% of UCLRs, both of which are higher than their respective averages of 25% and 33.1% of total UCLRs.

When rates of pitcher UCLRs by player year were compared, there were stark differences between warm-state freshmen and sophomores compared with cold-state freshmen and sophomores. Warm-state freshmen had UCLRs at significantly higher rates than cold-state freshmen and were 1.94 times more likely to undergo UCLR than cold-state freshmen. This observation was similar for sophomores, with significantly higher rates of warm-state sophomore UCLRs and a 2.04 times higher likelihood of having UCLRs compared with cold-state sophomores. In both warm and cold-state junior pitchers, there was no significant difference between incidence rates or incidence ratios.

Warm state senior pitchers demonstrated a similar trend to that of freshmen and sophomores. Although the observed rate difference between warm and cold seniors was lower compared with the rate differences observed in freshmen and sophomore age groups (0.002 vs 0.003 and 0.001, respectively), the incidence rate ratio was the highest of all age groups, meaning warm state seniors were 2.49 times more likely to undergo UCLR compared with cold state seniors. This result may be attributable to the lower instance of total UCLR during the senior year (29), or that warm state seniors have been exposed to these conditions for the longest period, contributing to potential wear and tear injury over time. This finding is significant, as previous studies have identified the weather as a potential risk factor for UCLR; however, no one has identified that younger college pitchers seem to be more susceptible to UCLRs in warm climates compared with cold climates.^13^

Previous studies have indicated that pitchers in the MLB who grew up pitching in warm weather climates underwent UCLR at a significantly higher rate than those who grew up pitching in cold weather climates.^13^ We sought to analyze whether this trend was similarly reflected in NCAA D1 athletes; thus, the climates where D1 pitchers played high school baseball were recorded and compared with respective pitcher years. Although no statistically significant differences were found in pitcher UCLRs versus their high school playing climate overall (*P* = .085), pitchers from warm environments were more likely than those from cold environments to sustain a UCLR while playing D1 baseball.

Underclassmen pitchers who played in warm high school climates had significantly higher rates of UCLR compared with cold high school climate pitchers. This finding further supports the calculated incidence rate ratios and suggests that pitching in warm temperatures may be associated with earlier instances of UCLR. In addition, upperclassmen pitchers from cold-climate high schools had significantly higher rates of UCLR than upperclassmen pitchers from warm-climate high schools. This might be attributable to warm climate pitchers, who may be more susceptible to UCL tears in their careers, either already sustaining and recovering from a previous UCLR or not yet being cleared to play after a recent UCLR. These results highlight that pitching in a warm temperature high school environment may be a significant risk factor for earlier incidence of UCLRs.

Another major risk factor identified for UCLR is increased pitching in adolescence.^10,20,26,27^ Age-appropriate injury prevention guidelines are available via Pitch Smart for UCL injury prevention in adolescent and college-level athletes.^21^ The guidelines state that in pitchers aged 19 to 22 years, there should be no more than a maximum of 120 pitches daily, followed by at least 5 days of rest following and a pitcher should only throw a maximum of 30 times in 1 day without needing a required rest day afterward. Until 2018, the NCAA had not formally enacted pitching maximums for pitchers, even though Pitch Smart guidelines were launched^7,9^ in 2014. The NCAA consistently failed to report pitcher counts for up to 33% of D1 games.^9,19^ In 2018, the NCAA implemented its pitching maximums, stating that players cannot pitch >110 pitches daily, followed by 3 days of rest.^7^ Although these guidelines are evidence-based and clearly outlined, UCLR rates in pitchers aged 15 to 19 years are higher than ever and increasing by 9% yearly.^3^

To mitigate UCL injuries and reconstruction surgeries, it is important for NCAA D1 baseball pitchers, parents, coaches, and trainers to manage pitchers’ cumulative, repetitive overhead motion via regulating practice and game frequency not just as college athletes but also as adolescents. Early education on the potential long-term effects of overuse injuries and the stricter implementation of formal pitching maximums may help prolong a pitcher’s throwing longevity and reduce the significant number of pitchers sustaining this career-altering injury. Further research should explore the physiological role that higher temperatures may play in UCL injury susceptibility in younger college pitchers. Injury prevention programs tailored individually to players’ pitching backgrounds should potentially be explored. With the increasing rates of UCLRs in pitchers everywhere, it is important for the NCAA and researchers to further investigate the potential link between weather and UCL injuries.

## Limitations

This study has several limitations. We did not adhere strictly to the 33rd parallel line to categorize data as being from warm versus cold states. For states bisected by the parallel, the entire area of the state was classified as warm. The UCLR NCAA D1 player injury data we utilized are not sponsored or sanctioned by the NCAA. The NCAA stresses that their data on player participation is self-reported from their D1 member institutions and as such may have errors that could impact their average pitchers per team calculations, which we utilized. We did not have access to the actual number of total NCAA D1 pitchers by academic year, which limited our analyses. In addition, the COVID-19 pandemic specifically affected college sports—especially baseball—in the spring of 2020, likely impacting the accuracy of both player injury counts and player participation rates. Because of the limited public information on minors, we were unable to discern whether pitchers sustained previous UCL injuries before college—a potential risk factor for both ipsilateral UCL retear and contralateral UCLR. The high school states of the injured pitchers were determined through internet searches, which does not account for pitchers who moved during high school or those who primarily pitched in a different climate before playing in high school.

## Conclusion

The data in this study suggest that NCAA D1 collegiate pitchers in warm states undergo UCLR at higher rates compared with pitchers in colder states. Warm state pitchers comprised more overall UCLRs in D1 programs, although they represent a smaller portion of total D1 baseball pitchers. In addition, warm-state pitchers underwent UCLR earlier in their college careers versus cold-state pitchers. Although the reported number of season games played between warm and cold D1 programs are not significantly different, increased pitcher UCLRs observed may be attributable to more games played during on-season and shorter recovery times in off-season due to favorable weather conditions or the physiologic effects of warm weather on tissue biomechanics.
